# Is There Decreasing Public Interest in Renal Transplantation? A Google Trends^TM^ Analysis

**DOI:** 10.3390/jcm9041048

**Published:** 2020-04-07

**Authors:** Andreas Kronbichler, Maria Effenberger, Jae Il Shin, Christian Koppelstätter, Sara Denicolò, Michael Rudnicki, Hannes Neuwirt, Maria José Soler, Kate Stevens, Annette Bruchfeld, Herbert Tilg, Gert Mayer, Paul Perco

**Affiliations:** 1 Department of Internal Medicine IV (Nephrology and Hypertension), Medical University Innsbruck, Anichstrasse 35, 6020 Innsbruck, Austria; christian.koppelstaetter@tirol-kliniken.at (C.K.); sara.denicolo@i-med.ac.at (S.D.); michael.rudnicki@i-med.ac.at (M.R.); hannes.neuwirt@i-med.ac.at (H.N.); gert.mayer@i-med.ac.at (G.M.); paul.perco@i-med.ac.at (P.P.); 2 Department of Internal Medicine I (Gastroenterology, Hepatology, Endocrinology and Metabolism), Medical University Innsbruck, Anichstrasse 35, 6020 Innsbruck, Austria; herbert.tilg@i-med.ac.at; 3 Department of Pediatrics, Yonsei University College of Medicine, 03722 Seoul, Korea; shinji@yuhs.ac; 4 Department of Pediatric Nephrology, Severance Children’s Hospital, Seoul 03722, Korea; 5 Institute of Kidney Disease Research, Yonsei University College of Medicine, Seoul 03722, Korea; 6 Department of Nephrology, Hospital Universitari Vall d’Hebron, Nephrology Research Group, Vall d’Hebron Research Institute (VHIR), 08035 Barcelona, Spain; mjsoler01@gmail.com; 7 Glasgow Renal and Transplant Unit, Queen Elizabeth University Hospital, Glasgow G51 4TF, UK; kate.stevens@glasgow.ac.uk; 8 Department of Clinical Sciences Interventions and Technology (CLINTEC), Division of Renal Medicine, Karolinska Institutet, Karolinska University Hospital, 171 77 Stockholm, Sweden; annette.bruchfeld@ki.se

**Keywords:** kidney transplantation, transplant numbers, live donors, public awareness, Google Trends^TM^

## Abstract

Background and objectives: Renal transplantation is the preferred form of renal replacement therapy for the majority of patients with end stage renal disease (ESRD). The Internet is a key tool for people seeking healthcare-related information. This current work explored the interest in kidney transplantation based on Internet search queries using Google Trends^TM^. Design, setting, participants, and measurements: We performed a Google Trends^TM^ search with the search term “kidney transplantation” between 2004 (year of inception) and 2018. We retrieved and analyzed data on the worldwide trend as well as data from the United Network for Organ Sharing (UNOS), the Organización Nacional de Trasplantes (ONT), the Eurotransplant area, and the National Health Service (NHS) Transplant Register. Google Trends^TM^ indices were investigated and compared to the numbers of performed kidney transplants, which were extracted from the respective official websites of UNOS, ONT, Eurotransplant, and the NHS. Results: During an investigational period of 15 years, there was a significant decrease of the worldwide Google Trends^TM^ index from 76.3 to 25.4, corresponding to an absolute reduction of −50.9% and a relative reduction by −66.7%. The trend was even more pronounced for the UNOS area (−75.2%), while in the same time period the number of transplanted kidneys in the UNOS area increased by 21.9%. Events of public interest had an impact on the search queries in the year of occurrence, as shown by an increase in the Google Trends^TM^ index by 39.2% in the year 2005 in Austria when a person of public interest received his second live donor kidney transplant. Conclusions: This study indicates a decreased public interest in kidney transplantation. There is a clear need to raise public awareness, since transplantation represents the best form of renal replacement therapy for patients with ESRD. Information should be provided on social media, with a special focus on readability and equitable access, as well as on web pages.

## 1. Introduction

Kidney transplantation is considered to be the optimal form of renal replacement therapy and has a positive impact on quality of life, survival rates of the recipients, and overall is considered cost-effective [1]. Due to organ shortage and longer waiting time, death on the waiting list is a serious concern and criteria for suitable organs have been extended. There are several advantages of live donor transplantation compared with deceased donor transplantation including lower risk of rejection, reduced waiting time for transplantation, and improved allograft and overall survival [2]. The frequency of live kidney donation is stable in the United States (US), while increasing in the Eurotransplant area and in the United Kingdom (UK) over the last 15 years. Despite these efforts there are currently 94,621 patients on the kidney waiting list in the US according to the United Network for Organ Sharing (UNOS), 10,791 (at the end of 2018) potential recipients in the Eurotransplant area and as of March 2019, approximately 5000 patients were waiting for a kidney transplant in the UK. Analysis of different surveys among the public revealed barriers towards live kidney transplantation [3], and strategies to overcome these barriers are necessary to increase the number of transplants.

Google Trends^TM^ generates data on spatial and temporal patterns according to specified keywords. A study comparing the reliability of Google Trends^TM^ in two settings, more common diseases with low media coverage and less common diseases with higher media coverage, found that Google Trends^TM^ seems to be influenced by media presence rather than by true epidemiological burden of one disease [4]. Several studies using Google Trends^TM^ data have been conducted recently. One of these investigated the influence of meteorological variables on relative search volumes for pain and found that selected local weather conditions were associated with online search volumes for specific musculoskeletal pain symptoms [5]. Analysis of Google Trends^TM^ search volume queries not only holds great promise in medicine, but also in other areas of research. Analysis of northern Europeans’ (Finland, Germany, Norway, Ireland, and the UK) web searching behavior on Mediterranean tourist destinations revealed a relationship between thermal conditions and the searching behavior, and the authors observed no time lag between the prevalence of thermal conditions and searching of the keywords [6].

In transplant medicine, public awareness is key to promote discussion around organ donation, both live and deceased. In the current study, we investigate the public interest in kidney transplantation using data on Internet search queries extracted from the Google Trends^TM^ tool.

## 2. Materials and Methods

### 2.1. Retrieving Transplantation Numbers for UNOS, ONT, and Eurotransplant

Data were retrieved by accessing the respective websites of the transplant organizations ((https://unos.org) for the UNOS, (http://www.ont.es) for the Organización Nacional de Trasplantes (ONT), (https://www.eurotransplant.org) for the Eurotransplant countries, and (https://www.nhsbt.nhs.uk) for the UK.

Information about live and deceased donor kidney transplantation over a period of 15 years (2004–2018) for the following countries was extracted from the web pages as stated above: United States of America (UNOS), Spain (ONT), Austria, Belgium, Croatia, Germany, Hungary, Slovenia, and the Netherlands (belonging to the Eurotransplant countries), and the UK (NHS Transplant Register).

### 2.2. Retrieving Google Trends^TM^ Data on Kidney Transplantation

The Google Trends^TM^ tool (https://trends.google.com/trends/) was used to retrieve data on Internet user search activities in the context of kidney transplantation. Google Trends^TM^ is a freely accessible tool that enables researchers to study trends and patterns of Google search queries [7]. It was implemented in 2004 and data on Internet search queries are available since then on a monthly basis. Google Trends^TM^ expresses the absolute number of searches relative to the total number of searches over the defined period of interest. The retrieved Google Trends^TM^ index ranges from 0 to 100, with 100 being the highest relative search term activity for the specified search query in any given month [7]. Thus, a search index of 50 indicates that the search activity for kidney transplantation was 50% of that seen at the time when search activity was most intense [7].

Worldwide Google Trends^TM^ indices were retrieved between January 2004 and December 2018 using the search term “kidney transplantation”. We retrieved Google Trends^TM^ indices for the US, Spain, the following European countries being part of the Eurotransplant network, namely Austria, Belgium, Croatia, Germany, Hungary, Slovenia, and the Netherlands, and the UK. No Google Trends^TM^ indices could be retrieved for Luxembourg. Whereas the worldwide search was performed in English, the individual searches in the respective countries were performed in the official languages (see Table S1).

### 2.3. Data Analysis

Annual average Google Trends^TM^ indices were calculated based on the monthly data downloaded from the Google Trends^TM^ webpage. Time-lag correlations between transplant numbers and Google Trends^TM^ indices were calculated using the ccf function of the tseries R package using a time lag between −3 and +3. The ggplot2 R package was used to generate all graphics. R version 3.4.1 was used for all analyses.

None of the queries in the Google database for this study can be associated to a particular individual. The database retains no information about the identity, Internet protocol address or specific physical location of any user. Furthermore, any original web search logs older than nine months are anonymized in accordance with Google’s privacy policy (www.google.com/privacypolicy.html).

## 3. Results

**Figure 1:**
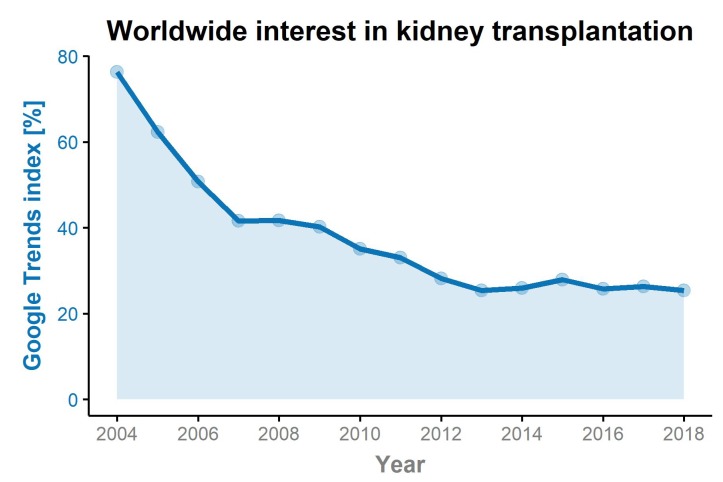
A worldwide decrease in the Google Trends^TM^ indices from inception to 2018 was found. During a period of 15 years, the index decreased from 76.3 to 25.4, corresponding to a change of −66.7%.

**Figure 2:**
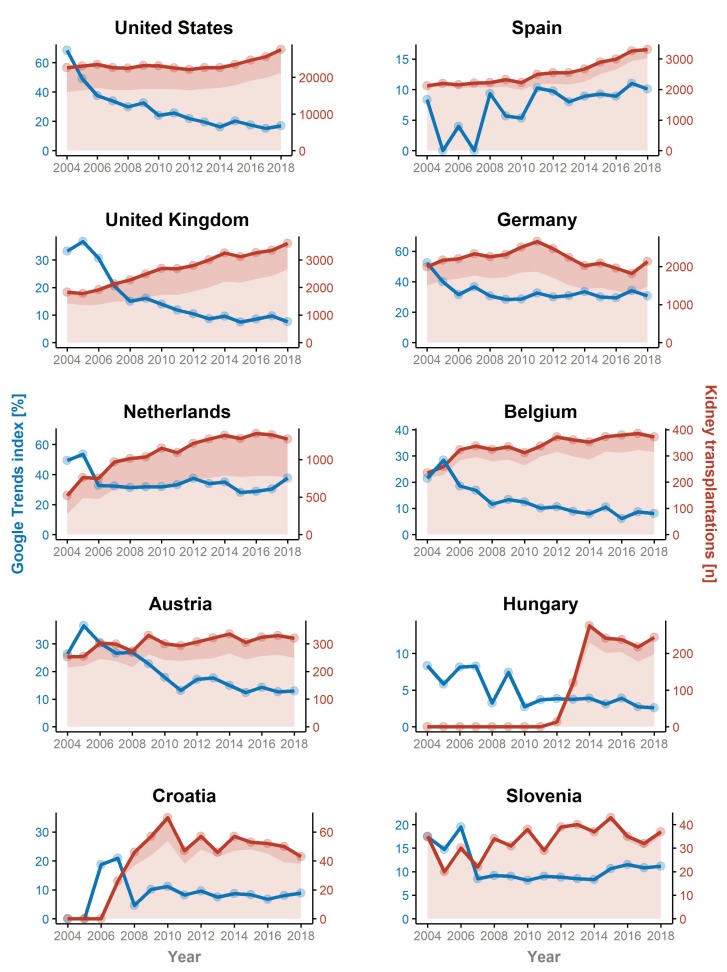
The respective numbers of renal transplants (red line) and the Google Trends^TM^ indices (blue line) are given for the United Nations of Organ Sharing (UNOS), the Organización Nacional de Trasplantes (ONT), the Eurotransplant areas, and the UK National Register. Numbers of deceased and living donor transplants are indicated by light and dark red areas. While there was a marginal increase in the Google Trends^TM^ index observed in Spain, the curves obtained from the UNOS, Eurotransplant areas, and the UK National Register mirror the worldwide trend.

The worldwide search query using Google Trends^TM^ highlighted a decrease from an index of 76.3 in 2004 to 25.4 in 2018 (absolute reduction −50.9, or a relative reduction of −66.7%, see Figure 1). This trend was particularly confirmed in the US, with a decrease of the Google Trends^TM^ index from an index of 68.4 to 17.0 (absolute reduction −51.4, relative reduction of −75.2%) over time. While an initial sharp decrease in search results was observed from an index of 68.4 to 37.6 (absolute reduction −30.8, relative reduction of −45.0%) within two years, there was a further decrease by 54.8% over the following thirteen years. In the same period of time, UNOS reported an increase of deceased donor kidney transplants from 16,007 in 2004 to 21,167 in 2018 (+32.2%); within the same period the live donor kidney transplantation rate remained stable (6648 in 2004 and 6442 in 2018, −3.1%). A similar search tendency of a decreased Google Trends^TM^ index was found for the Eurotransplant area and the UK. There was a modest increase in Google Trends^TM^ search queries in Spain, with a very low number in 2004 (index of 8.3) and 10.1 in 2018 (absolute increase +1.8, or a relative increase of +21.7%). In the same time-period the number of transplanted kidneys increased from 2125 to 3313 (+55.9%). In smaller countries, it is likely that events of interest to the public lead to an increase in search queries in that particular year. This for example might explain the increase in search queries in Austria in 2005 when a person of public interest received a second live-related kidney transplant in the same year. We observed an increase of Google Trends^TM^ search queries from an index of 26.3 in 2004 to 36.6 in 2005 (absolute increase +10.3 or relative increase of +39.2%). In the following years, a decrease was found with an index of 12.9 in 2018 (absolute reduction −13.4 or a relative decrease of −51.0%). Similar curves were observed in all Eurotransplant countries, even in countries with a higher number of live-related kidney transplants, for example, the Netherlands (48.1% in 2004 and 40.0% in 2018), where more web-based information retrieval might be expected. Online searches assessed by Google Trends^TM^ decreased from 49.3 to 37.8 (−11.5, or −23.3%) over 15 years. In Germany a decrease from 52.4 to 30.7 (−21.7, or −41.4%) was found in the same period, with even more pronounced reductions observed in Belgium (from 21.5 to 8.1, corresponding to a decrease of 13.4, or −62.3%) and Hungary (from 8.3 to 2.6, absolute reduction of −5.7 or relative reduction by −68.7%). In the UK, Google Trends^TM^ indices decreased from 33.25 to 7.58 with an absolute reduction of 25.67 and a relative reduction of −77.2%, mirroring the decrease observed in the US. An overview of Google Trends^TM^ changes over time and number of transplants (deceased donor and live donor transplantation) in the respective countries is highlighted in Table 1*,* Table S2, and Figure 2.

**Figure 3:**
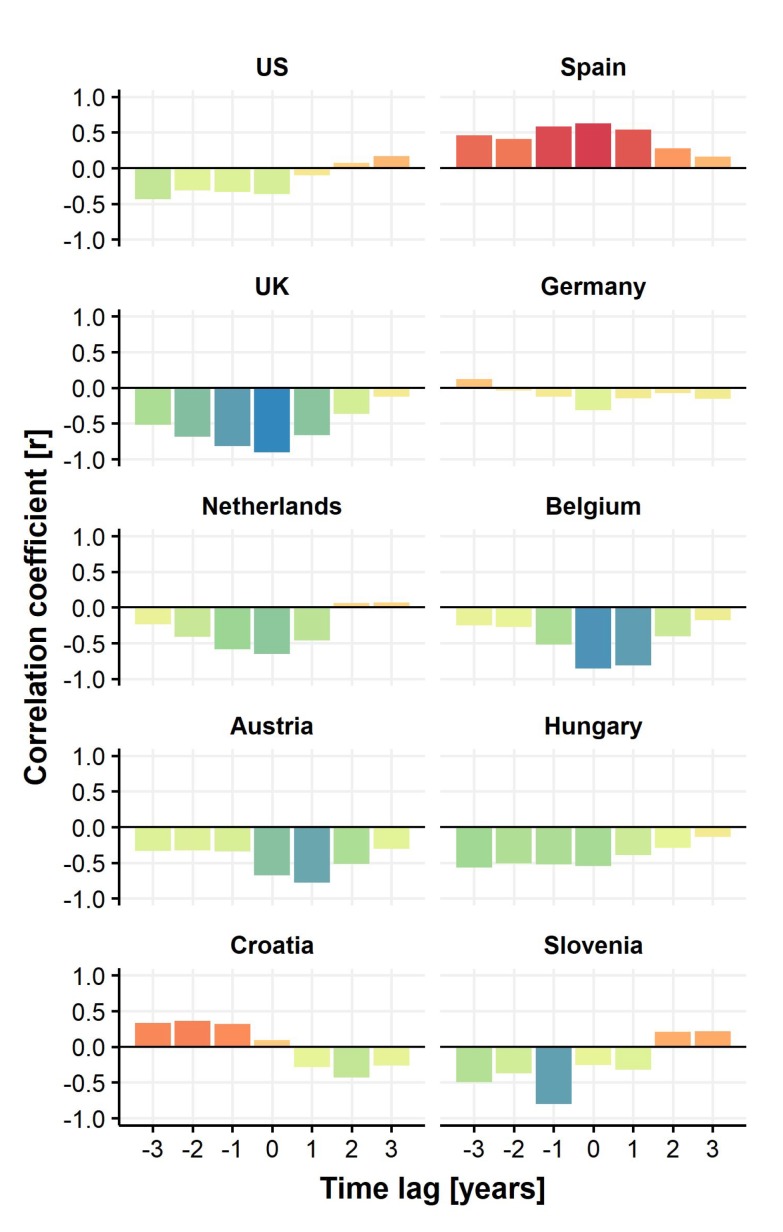
Time-lag correlations of Google Trends^TM^ indices and number of performed transplants for the countries under study. Negative correlations between Google Trends^TM^ indices and number of transplants are highlighted in green to blue whereas positive correlations are given in orange to red.

We used correlation analysis to compare the Google Trends^TM^ indices to the number of transplants over time and found negative correlations in particular for the UK, Belgium, and Austria, but also for Hungary, Slovenia, Germany, and the US. Spain is the only country where both transplant numbers as well as Google Trends^TM^ indices show positive correlations above 0.5 (Figure 3).

## 4. Discussion

To our knowledge, this is the first study investigating the trend of search queries for kidney transplantation. We observed a global decrease in public interest regarding kidney transplant, in particular in the UNOS, the Eurotransplant areas, and the UK. There is a global increase in transplanted kidneys, however, an increase in waiting time and a shortage of kidney donors highlight the demand [8]. Kidney transplant is the optimal form of renal replacement therapy for patients with end stage renal disease, improving both quality and quantity of life. Whilst this is true for both live and deceased organ donation, recipients of a live donor kidney transplant demonstrate better outcomes at both, one and five-years post transplantation [9]. Thus, raising and maintaining awareness about kidney transplants is imperative. How can we achieve this essential goal? Along with strategies discussed below, supra-national alliances such as the European Kidney Health Alliance (EKHA) are essential.

Efforts should be made to increase the number of live kidney donor transplants which are performed [9]. To help overcome hurdles like lack of awareness, particularly in populations with lower rates of live donor kidney transplants, namely ethnic minority populations and in groups who suffer from socioeconomic deprivation [10,11], successful campaigns have been orchestrated using both traditional media as well as online media, and community-based venues. By using Google Analytics^TM^, the authors found an eight-fold increase in traffic to the Infórmate website, a website developed by the Northwestern University faculty in partnership with the National Kidney Foundation, compared to the pre-campaign period [12]. Website exposure was associated with a significant knowledge score increase between pretest and posttest assessments, which was maintained at a follow-up assessment at three weeks [13]. Readability and accessibility of online living donor and deceased donor recipient material is essential. An analysis of the top ten websites for both revealed that the reading level for the living donor materials was 12.54, while it was 12.87 for the deceased donor materials, corresponding to a university level. Overall, the readability of online material remains too high for the corresponding health literacy rates among potential kidney transplant recipients [14]. Whilst the readability must be increased, Information Score (IS) assessment also revealed a poor quality of many websites and that more input from transplant physicians is needed. Information should be freely available in multiple languages, as well as in Braille format and as audio text. Generally, websites belonging to academic institutions have higher IS than professional, or commercial websites [15]. Among 46 Italian YouTube^®^ videos analyzed for usefulness to inform about live donor kidney transplantation, only a minority (15.2%) were categorized to contain useful information for the general population [16].

Kidney transplant knowledge should be improved in potential recipients. The Knowledge Assessment of Renal Transplantation (KART) contains 15 items including basic information about the procedure, prognosis, and insurance issues, and has an acceptable evidenced reliability. The KART distinguished patients who spent more or less than one hour receiving different types of education, including communication between doctors and medical staff, reading brochures, browsing the Internet, and watching videos [17]. Limited knowledge is not only present among patients but is also evident amongst medical students. In total, 96% were aware of the possibility of live donor kidney transplantation, but only 8% of the surveyed students were registered as potential donors in this South African study [18]. Similarly, a study from Leeds found that students had a basic understanding of organ donation and transplantation but lack detailed knowledge, such as understanding the criteria which are commonly used for brain death testing [19]. A study from India reporting on 200 interviews found that awareness will promote organ donation and there is a need for effective campaigns that educate people with relevant information, since a majority (59%) believed that donated organs might be misused, abused, or misappropriated [20].

Potential kidney transplant donors and recipients and those who have donated or received a transplant should be invited to share their experience online, in person, and on social media platforms. A survey involving 199 patients revealed that half use social media (52.3%, not further specified which channels were used) and most reported to be willing to post information about live kidney donation on their social networks (51%) [21]. Renal patients’ organizations must also be supported and encouraged to provide information via social media.

Transplant physicians, surgeons, and nursing staff may also use social media to increase awareness of kidney transplantation. A survey among members of the American Society of Transplant Surgeons indicated that among 299 physicians who completed the survey, 59% use social media to communicate with surgeons, 57% with transplant professionals, 21% with transplant recipients, 16% with living donors, and 15% with waitlisted candidates. Younger age and fewer years of experience in transplantation were significantly associated with a stronger belief that social media may be influential in living organ donation [22].

Religious differences in mixed communities may play a role. In a Dutch study, the impact of religion on live donor kidney transplantation was assessed. The authors reported that religion is not perceived as an obstacle to live donation in the Netherlands. However, there is a necessity for increased clarity and awareness for different religions with respect to live donation [23]. While most of the patients seemed to favor live donor kidney transplantation, a variety of potentially modifiable barriers were identified, including inadequate patient education, emotional factors, restrictive social influences, and suboptimal communication [24].

Altruistic live donation will play an increasing role in the future. Social media is used to facilitate transplantation (i.e., through websites such as MatchingDonors.com), which was implemented as early as 1994. An organ registration fee is one of the ethical concerns of such strategies. Facebook and Twitter are freely available platforms to communicate with others within groups and via hashtags and offer the opportunity to connect with potential live donors [25]. Moorlock et al. critically assessed the so-called “identifiable victim effect” and proposed that institutionally organized personal case-based campaigns aimed at promoting specific recipients for directed donation, despite its ethical concerns, should be preferred to facilitate altruistic live donation [26]. Building a framework for social media and organ donation is necessary and recommendations for transplant hospitals have been issued [27]. Programs such as the Kidney Coach Program (KCP) need to be implemented in the clinical practice to equip individuals (candidates and advocates for candidates) with tools to identify potential donors, which enables individuals to discuss donation with people in their social network [28].

Amongst countries participating in the Eurotransplant program, different legal strategies are employed; for instance, in Germany a potential donor needs to declare willingness to be registered as a deceased donor or ‘opt in’ [29]. This can increase the time it takes to ascertain suitability and thus delay transplant surgery. It also means that there are likely to be many willing donors, who simply do not register but if the system were ‘opt out’ would be very willing to be organ donors. Furthermore, ‘opt in’ systems for deceased donor donation lead to ongoing political debate which one might anticipate would help to raise awareness. When this was assessed via a Google Trends^TM^ search, the decrease in the Google Trends^TM^ index mirrored the changes observed in other Eurotransplant countries and thus this ongoing debate did not influence the public interest as assessed by Google Trends^TM^. By the end of 2020, the organ donation laws in the UK will have moved from an ‘opt in’ system for deceased donor organ donation to an ‘opt out’ system (i.e., a deemed authorization system, applicable to the vast majority of the population with some notable exceptions). Northern Ireland is excepted from this change and the donation system there remains ‘opt in’ [30]. A significant factor in this change in legislation is the result of campaigning and lobbying from a nine-year old boy, Max Johnson, and his family. Max was awaiting a heart transplant which he ultimately received from Keira Ball, a nine-year old girl whose parents selflessly agreed to donate her organs. The legislation is to be commonly referred to as Max and Keira’s Law [31].

Whilst this study shows a decreasing interest in web-based information over time in most areas, the number of live kidney donations increased in the ONT, the Eurotransplant areas, and the UK, while it was almost stable over time in the UNOS area (−3.1% from 2004 to 2018). This highlights that in most countries information from the treating physicians is more important than from the World Wide Web. A scoping review addressed strategies to increase live kidney donation and found that recipient-based education that reaches friends and family has the best evidence of being effective [32]. In contrast to the global trend, the Google Trends^TM^ search highlighted an increase in search queries in Spain. In the same time period, the number of live and deceased kidney transplantation increased by 480.3% and 46.3%. It is tempting to speculate that either a sharp increase in transplant numbers or the implementation of non-heart-beating donation increased public interest [33].

This study has a few limitations. While Google Trends^TM^ captures Google search queries and might act as a surrogate for public interest, Google is not the only available search engine next to other social media networks being used to search for information on the Internet. Previous work by others however indicates that Google Trends^TM^ is a very valid measure of public interest. Additionally, the results obtained from Google Trends^TM^ represent only relative numbers with no information on the absolute interest being available. We restricted our analysis to countries with an excellent documentation of transplant numbers and excluded countries from Asia and Africa, although they were included in the worldwide Google Trends^TM^ analysis.

In conclusion, our Google Trends^TM^ analysis found a decreasing public interest in renal transplantation. Strategies to inform the general population about unmet needs in the transplant setting (i.e., reduction of the waiting list time and live kidney donation) need to be utilized by all involved in the care of patients with kidney disease, by the patients themselves, and by national societies and academic institutions. Easily accessible information must be provided which is coherent and available in multiple languages including Braille and audio text. The message conveyed should be consistent and the information should be made available on multiple platforms including webpages, social media, and paper format. This may help reduce barriers in accessing information for different groups and improve outcomes according to the principles of patient-centered care.

## Figures and Tables

**Table 1:** The respective year, number of search queries using Google Trends^TM^, and the total number of kidney transplantations performed (deceased donor and living donor).

Year.	World GT.	US GT.	US Tx.	UK GT.	UK Tx.	ESP GT.	ESP Tx.	B GT.	B Tx.	NL GT.	NL Tx.	GER GT.	GER Tx.	AUT GT.	AUT Tx.	SLO GT.	SLO Tx.	H GT.	H Tx.	CRO GT.	CRO Tx.
**2004**	76.3.	68.4	22,655	33.25	1836.	8.3	2125	21.5	235	49.3	520	52.4	1991	26.3	253	17.4	35	8.3	0	0.0	0
**2005**	62.3	49.1	23,057	36.75	1783.	0.0	2200	28.4	260	53.5	762	40.0	2165	36.6	255	14.8	20	5.8	0	0.0	0
**2006**	50.8	37.6	23,530	30.67	1915.	4.0	2157	18.6	324	32.8	752	31.5	2206	30.3	303	19.5	30	8.2	0	18.8	0
**2007**	41.6	33.8	22,677	20.58	2130.	0.0	2211	16.9	338	32.4	968	36.7	2336	26.6	299	8.5	22	8.3	0	21.0	26
**2008**	41.8	29.8	22,489	15.00	2282.	9.3	2229	11.6	325	31.3	1012	30.8	2257	26.9	273	9.2	34	3.3	0	4.7	46
**2009**	40.3	32.6	23,216	16.08	2495.	5.7	2328	13.3	335	31.8	1036	28.4	2317	22.8	331	9.0	31	7.4	0	10.2	57
**2010**	35.1	24.1	23,178	14.00	2694.	5.3	2225	12.4	313	31.8	1151	28.7	2512	17.8	300	8.2	38	2.8	0	11.2	70
**2011**	33.1	25.7	22,589	11.83	2686.	10.3	2498	10.1	338	33.3	1091	32.6	2660	13.2	293	9.0	29	3.7	0	8.3	47
**2012**	28.2	21.9	22,106	10.50	2799.	9.8	2551	10.6	373	37.5	1215	30.0	2471	17.2	307	8.8	39	3.8	13	9.6	57
**2013**	25.4	19.6	22,629	8.67	3001.	8.0	2552	8.9	361	33.9	1273	30.8	2241	17.7	321	8.5	40	3.8	120	7.5	46
**2014**	25.9	16.2	22,646	9.58	3259.	8.9	2678	8.0	353	35.1	1321	33.5	2021	15.0	336	8.3	37	3.9	276	8.7	57
**2015**	27.9	20.2	23,506	7.50	3121.	9.3	2905	10.5	374	28.2.	1280.	30.0	2089	12.3	305	10.7	43	3.1	242	8.3	53
**2016**	25.8	17.6	24,689	8.50	3268.	8.9	2997	6.1	380	28.8	1348	29.5	1957	14.3	324	11.5	35	3.9	238	6.8	52
**2017**	26.3	15.1	25,660	9.75	3351.	11.0	3269	8.7	386	30.5	1329	34.2	1814	12.7	330	10.8	32	2.8	218	8.0	50
**2018**	25.4	17.0	27,609	7.58	3608.	10.1	3313	8.1	373	37.8	1274	30.7	2130	12.9	320	11.2	37	2.6	244	8.8	43
**Change (%).**	−66.7	−75.1	+21.9.	−77.2	+196.5	+21.7	+55.9	−62.3	+58.7	−23.3	+245.0	−41.4	+7.0	−51.0	+26.5	−35.6	+5.7	−68.7	-	-	-

Abbreviations: GT (Google Trends^TM^), US (United States of America), UK (United Kingdom), ESP (Spain), B (Belgium), NL (the Netherlands), GER (Germany), AUT (Austria), SLO (Slovenia), H (Hungary), CRO (Croatia), Tx. (transplants).

## Supplementary Materials

The following are available online at https://www.mdpi.com/2077-0383/9/4/1048/s1, Table S1: Search terms for the respective countries and languages are given, Table S2: The numbers of deceased donor and living donor kidney transplantations over time are given.
