# Supplementary material for: Is There Decreasing Public Interest in Renal Transplantation? A Google TrendsTM Analysis
Source: J Clin Med. 2020 Apr 7;9(4):1048. doi: 10.3390/jcm9041048 (PMC7231300; doi:10.3390/jcm9041048)
Supplement: Supplementary file 1 [file jcm-09-01048-s001.pdf]

# Supplementary Material: Is There Decreasing Public Interest in Renal Transplantation? A Google Trends™ Analysis

**Andreas Kronbichler, Maria Effenberger, Jae Il Shin, Christian Koppelstätter, Sara Denicolò, Michael Rudnicki, Hannes Neuwirt, Maria Soler, Kate Stevens, Annette Bruchfeld, Herbert Tilg [2], Gert Mayer and Paul Perco**

**Table S1.** Search terms for the respective countries and languages are given.

| *Country* | *Language* | *Search term* |
|---|---|---|
| *United States (US)* | English | kidney transplantation |
| *United Kingdom (UK)* | English | Kidney transplantation |
| *Spain (ESP)* | Spanish | trasplante de riñón, transplante renal |
| *Belgium (B)* | French | transplantation rénale |
| *Luxembourg (LUX)* | French | transplantation rénale |
| *Netherlands (NL)* | Dutch | niertransplantatie |
| *Germany (GER)* | German | Nierentransplantation |
| *Austria (AUT)* | German | Nierentransplantation |
| *Slovenia (SLO)* | Slovenian | presaditev oder transplantacijo ledvic |
| *Hungary (H)* | Hungarian | veseátültetés |
| *Croatia (CRO)* | Croatian | transplantacija bubrega |

**Table S2.** The numbers of deceased donor and living donor kidney transplantation over time are given.

| Year | USdec | USliv | UKdec | UKliv | ESPdec | ESPliv | Bdec | Bliv | NLdec | NLliv | GERdec | GERliv | AUTdec | AUTliv | SLOdec | SLOliv | Hdec | Hliv | CROdec | CROliv |
|---|---|---|---|---|---|---|---|---|---|---|---|---|---|---|---|---|---|---|---|---|
| **2004** | 16,007 | 6,648 | 1,424 | 412 | 2,064 | 61 | 216 | 19 | 270 | 250 | 1,502 | 489 | 215 | 38 | 35 | 0 | 0 | 0 | 0 | 0 |
| **2005** | 16,485 | 6,572 | 1,361 | 422 | 2,113 | 87 | 228 | 32 | 487 | 275 | 1,643 | 522 | 220 | 35 | 20 | 0 | 0 | 0 | 0 | 0 |
| **2006** | 17,095 | 6,435 | 1,376 | 539 | 2,055 | 102 | 283 | 41 | 474 | 278 | 1,684 | 522 | 246 | 57 | 30 | 0 | 0 | 0 | 0 | 0 |
| **2007** | 16,634 | 6,043 | 1,491 | 639 | 2,074 | 137 | 296 | 42 | 608 | 360 | 1,769 | 567 | 237 | 62 | 22 | 0 | 0 | 0 | 26 | 0 |
| **2008** | 16,521 | 5,968 | 1,505 | 777 | 2,073 | 156 | 280 | 45 | 601 | 411 | 1,692 | 565 | 216 | 57 | 34 | 0 | 0 | 0 | 37 | 9 |
| **2009** | 16,829 | 6,387 | 1,641 | 854 | 2,093 | 235 | 286 | 49 | 619 | 417 | 1,717 | 600 | 262 | 69 | 31 | 0 | 0 | 0 | 44 | 13 |
| **2010** | 16,900 | 6,278 | 1,731 | 963 | 1,985 | 240 | 264 | 49 | 678 | 473 | 1,847 | 665 | 241 | 59 | 38 | 0 | 0 | 0 | 54 | 16 |
| **2011** | 16,816 | 5,773 | 1,732 | 954 | 2,186 | 312 | 298 | 40 | 651 | 440 | 1,865 | 795 | 238 | 55 | 29 | 0 | 0 | 0 | 38 | 9 |
| **2012** | 16,487 | 5,619 | 1,862 | 937 | 2,190 | 361 | 316 | 57 | 730 | 485 | 1,705 | 766 | 244 | 63 | 39 | 0 | 13 | 0 | 48 | 9 |
| **2013** | 16,896 | 5,733 | 2,004 | 997 | 2,170 | 382 | 298 | 63 | 753 | 520 | 1,516 | 725 | 248 | 73 | 40 | 0 | 102 | 18 | 43 | 3 |
| **2014** | 17,108 | 5,538 | 2,224 | 1035 | 2,255 | 423 | 286 | 67 | 787 | 534 | 1,401 | 620 | 265 | 71 | 37 | 0 | 230 | 46 | 47 | 10 |
| **2015** | 17,878 | 5,628 | 2,177 | 944 | 2,517 | 388 | 317 | 57 | 767 | 513 | 1,444 | 645 | 243 | 62 | 43 | 0 | 202 | 40 | 48 | 5 |
| **2016** | 19,060 | 5,629 | 2,321 | 947 | 2,654 | 343 | 313 | 67 | 784 | 564 | 1,360 | 597 | 257 | 67 | 33 | 2 | 204 | 34 | 45 | 7 |
| **2017** | 19,849 | 5,811 | 2,423 | 928 | 2,937 | 332 | 323 | 63 | 778 | 551 | 1,257 | 557 | 261 | 69 | 30 | 2 | 178 | 40 | 39 | 11 |
| **2018** | 21,167 | 6,442 | 2,668 | 940 | 3,020 | 293 | 316 | 57 | 764 | 510 | 1,492 | 638 | 250 | 70 | 35 | 2 | 199 | 45 | 38 | 5 |
| **Change (%)** | +32.2 | -3.1 | +187.4 | +228.2 | +46.3 | +480.3 | +46.3 | +300 | +283.0 | +204.0 | -0.7 | +30.5 | +16.3 | +84.2 | +/-0 | - | - | - | - | - |

Abbreviations: US (United States of America), dec (deceased), liv (living), UK (United Kingdom), ESP (Spain), B (Belgium), NL (The Netherlands), GER (Germany), AUT (Austria), SLO (Slovenia), H (Hungary), CRO (Croatia).
